# Effect of Kinesiology Tape on Muscle Activation of Lower Extremity and Ankle Kinesthesia in Individuals With Unilateral Chronic Ankle Instability

**DOI:** 10.3389/fphys.2021.786584

**Published:** 2021-12-17

**Authors:** Lulu Yin, Kun Liu, Chengmei Liu, Xiaodong Feng, Lin Wang

**Affiliations:** ^1^Rehabilitation Center, The First Affiliated Hospital of Henan University of Traditional Chinese Medicine, Zhengzhou, China; ^2^Department of Rehabilitation Medicine, Shanghai Jiao Tong University Affiliated Sixth People’s Hospital, Shanghai, China; ^3^College of Rehabilitation Medicine, Henan University of Chinese Medicine, Zhengzhou, China; ^4^School of Kinesiology, Shanghai University of Sport, Shanghai, China

**Keywords:** kinesiology tape, chronic ankle instability, electromyography activity, computerized dynamic posturography, kinesthesia

## Abstract

**Background:** The purpose of the study was to determine the effect of kinesiology tape (KT) on lower limb muscle activation during computerized dynamic posturography (CDP) tasks and ankle kinesthesia in individuals with chronic ankle instability (CAI).

**Methods:** Thirty-five men with CAI participated in this study. The experimental procedure followed a repeated measures design. Muscle activation of lower extremity and ankle kinesthesia of participants were measured using four taping treatments, namely, KT, athletic tape (AT), sham tape (ST), and no tape (NT) in a randomized order. Muscle activation was assessed using surface electromyography (sEMG) synchronized with CDP tests from seven lower extremity muscles of the unstable limb. Ankle kinesthesia was measured by using a threshold to detect the passive motion direction of the unstable ankle. Parameters were analyzed by using a one-way repeated measures ANOVA and followed by pairwise comparisons with a Bonferroni correction.

**Results:** No significant difference was observed among different taping treatments for the majority of parameters during CDP. Except for condition 4 with open eyes, sway-referenced surface, and fixed surround in the sensory organization test (SOT), gastrocnemius medialis root mean square (RMS) was 28.19% lower in AT compared with NT (*p* = 0.021, 95% CI = 0.002–0.039), while gastrocnemius lateralis RMS was 20.25% lower in AT compared with KT (*p* = 0.038, 95% CI = 0.000–0.021). In forward-small sudden translation from motor control test (MCT), for peroneal longus (PL), RMS was 24.04% lower in KT compared with ST (*p* = 0.036, 95% CI = 0.000–0.018). In toes-down sudden rotation from adaption test (ADT), for PL, RMS was 23.41% lower in AT compared with ST (*p* = 0.015, 95% CI = 0.002–0.027). In addition, no significant difference was observed for a threshold to the detection of passive motion direction among different taping treatments.

**Conclusion:** This study indicated that KT had minimal effect on the muscle activation of the unstable lower limb during static stance, self-initiated, and externally triggered perturbation tasks from CDP and ankle kinesthesia among individuals with CAI, suggesting that the benefit of KT was too small to be clinically worthwhile during application for CAI.

## Introduction

An ankle sprain is one of the most common musculoskeletal injuries associated with soccer, basketball, and volleyball (Attenborough et al., 2014). Given that it is generally considered self-coped and standardized rehabilitation is ignored, large proportions of patients develop frequently a cluster of symptoms, including persistent ankle pain, swelling, and a feeling of “giving away.” These symptoms are characteristics of chronic ankle instability (CAI), a multifaceted disorder that includes the destruction of ligament integrity, limited range of motion, decreased postural control (Delahunt et al., 2018), decreased peroneal muscle capacity (Khalaj et al., 2020), and proprioception deficits (Thompson et al., 2018; Xue et al., 2021). Consequently, CAI results in physical and economic burden, lower level of physical activity, reduced quality of life (Gribble et al., 2016), and increased risk of ankle osteoarthritis. Therefore, prevention and treatment of CAI are major challenges, and physical therapy plays a vital role in the management of CAI. Current evidence-based management of CAI involves non-steroidal anti-inflammatory drugs, early mobilization, balance training, bracing, and taping (Doherty et al., 2017; Kosik et al., 2017).

Kinesiology tape (KT), a water-resistant and elastic tape that could be stretched longitudinally to 90–140% of the initial length (Kase et al., 2003), has been used in the prevention and treatment of CAI (Williams et al., 2012; Kamper and Henschke, 2013; Lim and Tay, 2015) due to its cheap price, non-invasive, safe, and convenient properties. Previous research assumed its mechanism of action to include reduced pain and improved proprioception through stimulation of sensory afferents (Liu et al., 2020a,b), enhanced lymphatic reflux through increased subcutaneous tissue space (Lietz-Kijak et al., 2018), and prompt soft tissue structural stability (Kamper and Henschke, 2013). In addition, KT may facilitate muscle activation while applying from origin to insertion of the muscle according to manufacturers (Kase et al., 2003). However, the results on the effectiveness of KT for sports injury management are inconsistent (Williams et al., 2012; Lim and Tay, 2015; Nunes et al., 2015).

Proprioception, which is defined as the perception of position and movement, is involved in joint position sense, kinesthesia, force sense, and vibration (Xue et al., 2021). Only a few studies investigate the influence of KT on the proprioception of individuals with CAI. Yu et al. (2021) found that KT can be used to improve ankle inversion proprioceptive performance during landing for CAI participants. Simon et al. (2014) also revealed that KT could reduce the error of force sense of ankle eversion. However, some studies (Halseth et al., 2004; Bailey and Firth, 2017) did not support the use of KT as a method for improving proprioception. Therefore, the effect of KT on ankle proprioception during CAI remained unclear. At present, no study has explored the effect of KT on the proprioception of individuals with CAI from the perspective of kinesthesia. Compared with joint position sense and force sense, which require active contraction of muscles, kinesthesia judges motion generation when a joint moves at a very low speed at 0.4–2°/s. Kinesthesia tests slow-adapting mechanoreceptors and reduces the contribution of the muscle spindle and Golgi tendon organs to the joint movement (Winter et al., 2015; Han et al., 2016). Due to the damaged muscles and ligaments of CAI (Xue et al., 2021), it was important to analyze the function of KT on ankle joint kinesthesia.

Moreover, the current relevant study about the application of KT on the lower limb muscle activation among individuals with CAI is limited, and the results are controversial. It has been demonstrated that acute application of KT could decrease the velocity of the frontal plane sway and peroneus longus activity magnitude (Sarvestan et al., 2020). Ataullah et al. (2021) also confirmed that KT was effective in significantly decreasing the electromyography (EMG) activity of gluteus medius during a single-leg squat for CAI. Similarly, KT was proved to shorten the duration of gastrocnemius activity during gait (Martínez-Gramage et al., 2016). Conversely, Briem et al. (2011) found that KT did not alter muscle activation in healthy male participants before, during, and after a sudden inversion perturbation. Kodesh and Dar (2015) concluded that KT did not contribute to fibularis longus muscle activation following ankle muscle fatigue among individuals with CAI. This controversy in the outcomes put an emphasis on the importance of precise investigations on the impact of ankle KT on muscular activities during CAI.

However, a previous study investigated muscle activation in individuals with CAI mostly from result-oriented tasks such as the star excursion balance test (Labanca et al., 2021). Hence, these studies could not distinguish the proportion of vision, proprioception, and vestibular sensation in integrated postural control maintenance. Only a few studies evaluated the muscle activation of individuals with CAI through a method that combines EMG with computerized dynamic posturography (CDP) (Alguacil-Diego et al., 2018; Yin and Wang, 2020; Molina-Rueda et al., 2021). CDP is an instrument that consists of movable visual surround and support surfaces, providing a variety of visual, somatosensory, and vestibular self-initiated perturbations during sensory organization test (SOT) (De-La-Torre-Domingo et al., 2015). In addition, CDP includes static postural control test and unexpected externally triggered perturbations with different support surface motor modes (translation, rotation) that attempt to disrupt body equilibrium and displace the total body center of mass. It could provide real-time and quantifiable sensory feedback, simulate daily life, which could be faced by CAI patients with unexpected interference to some extent (Molina-Rueda et al., 2021). Considering the postural control tasks involved in CDP, understanding the effect of KT on the activation of lower limb muscles is important to explore sensorimotor coordination.

Therefore, this study aimed to explore the effect of KT on muscle activation of the lower extremity during CDP system tasks and kinesthesia in individuals with CAI. The significance of this study was to provide a more evidence-based basis and practical reference for the clinical application of KT in the CAI population. We hypothesized that KT could provide a positive effect for enhancing the activity of lower limb muscles and ankle kinesthesia.

## Materials and Methods

### Participants

Considering a power of 0.90, a level of 0.05 in repeated measures ANOVA, and a dropout rate of 0.15, a minimum of 31 participants were required. Finally, 35 male college students (age: 22.97 ± 2.81 years; height: 1.78 ± 0.06 cm; weight: 73.49 ± 12.33 kg; BMI: 23.27 ± 3.55 kg/m^2^) volunteered to participate in this study. The inclusion criteria were (1) male college students, regular daily activity (professional athletes or sedentary men were not included); (2) have a history of at least 1 significant ankle sprain, and the initial sprain occurred at least 12 months before the study enrollment; (3) feelings of “giving away” of the injured ankle joint and/or recurrent sprain and/or “feeling of instability”; and (4) a score of less than 24 in the Cumberland Ankle Instability Tool questionnaire (Gribble et al., 2014). Participants who had a history of bilateral sprains, lower limb fracture, operation, nervous and vestibular system disease, or allergic to taping were excluded. All participants were educated about the related risks of injury and signed an informed consent form. This study was approved by the Ethics Committee of the Shanghai University of Sport.

### Taping Procedure

**FIGURE 1:**
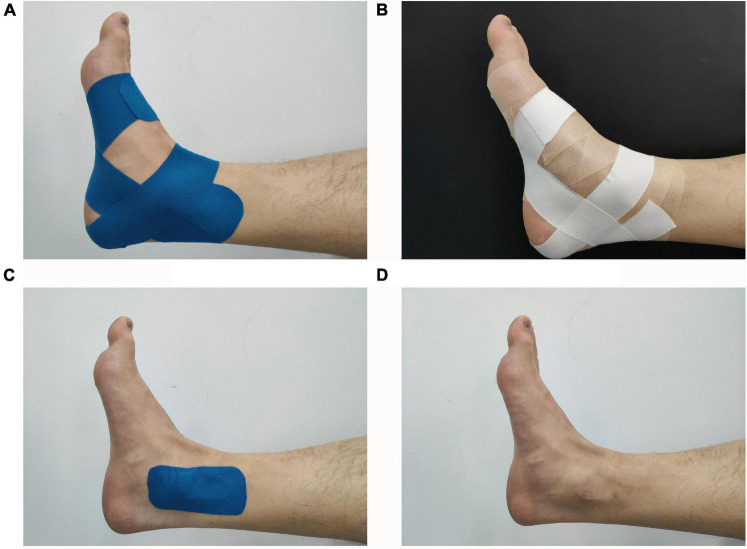
Different ankle taping treatments. **(A)** Kinesiology tape, **(B)** athletic tape, **(C)** sham tape, and **(D)** no tape.


The taping area should be clear of hair and wiped with alcohol before taping. Each participant received the following treatments randomly: real taping: KT (50 mm × 5 m) with 50% tension (De-La-Torre-Domingo et al., 2015); athletic tape (AT) (50 mm × 13 m), which aimed to find out if the material of tape would affect performance (Briem et al., 2011; Sarvestan and Svoboda, 2020); control taping: sham tape (ST), which judged the placebo effect of taping (Sawkins et al., 2007; Mak et al., 2019); and no taping (NT) (Yin and Wang, 2020). For KT and AT, we selected ankle balance taping (ABT) methods, which had been shown to provide support for the tibiotalar joint and ligaments of the medial and lateral malleolus (Kim and Shin, 2017; Lee and Lee, 2017; Figure 1). To maintain the consistency of the KT tension applied to the ankle joint, the ankle-length was considered when calculating the KT length using Eq. 1 (Yam et al., 2019; Wei et al., 2020).

(1) $$\mathrm{Actual}\mathrm{length}\mathrm{of}\mathrm{tape}\mathrm{to}\mathrm{cut}\left(\mathrm{cm}\right)=\left[\left(\frac{x-4}{1.5}\right)+4\right]\times 1.10$$

where *x* is the measured length between the origin and insertion sites. The ankle length was set at 4 cm (2 cm each for proximal and distal sites). For ST, a strip of KT without tension was adhered to the medial and lateral malleolus to avoid cutaneous input (Sawkins et al., 2007; Lee and Lee, 2017). This technique attempts to evaluate the placebo effect. One experienced therapist accomplished all taping tasks to relieve the operator’s deviation. Participants were not told about the function of various treatments. The order of taping was counterbalanced randomized, which was suitable for experiments conducted using repeated measures design (Supplementary Material 1). It aimed to reduce the chances of the order of treatment, influencing the results (Liu et al., 2020a). A wash-out period of 1 week was set to relieve any learning effect (Bailey and Firth, 2017).

### Outcome Measures

The measurement in this study included two parts, namely, muscle activation during CDP tasks and ankle kinesthesia.

#### Muscle Activation Instrument

A surface electromyography (sEMG) system (Myon AG, Schwarzenberg, Switzerland) with pairs of bipolar Ag/AgCl surface electrodes (Ambu Blue Sensor N-Electrodes, Denmark) was used. Skin preparation and electrode placement followed the guidelines (Hermens et al., 2000). We selected seven muscles: vastus medialis (VM), vastus lateralis (VL), biceps femoris (BF), tibialis anterior (TA), peroneal longus (PL), gastrocnemius medialis (GM), and gastrocnemius lateralis (GL) in the unstable side of the lower extremity. EMG raw data were collected at a sampling rate of 1,000 Hz.

Participants were instructed to wear a harness loosely and stand barefoot on two platforms (23 cm × 46 cm) of the CDP system SMART EquiTest Mode (Version 9.3, Copyright© 1989–2016 Natus Medical Incorporated). The CDP system consists of a movable platform and visual surround. The EMG system was synchronized in time with the CDP system *via* a wire. Once the CDP was triggered, the EMG signals would acquire automatically in the following tasks. Before each task, all participants were initially familiarized with CDP by an attempt with instruction. The order of tasks was randomized to avoid the order effect.

##### Sensory Organization Test

The SOT evaluated the integration of sensory information by selectively interfering with the visual, somatosensory, and vestibular systems. The interference was sway-referenced, which meant that the tilt of the platform and/or visual surround directly followed the participant’s anterior–posterior sway. This test required participants to stand upright to keep their center of gravity (COG) as steady as possible to cope with six different conditions described in Table 1. Each trial lasted for 20 s and was repeated three times in succession.

**TABLE 1:** Sensory organization test.

Condition	Eyes	Surface	Surround	Interference
1	Open	Fixed	Fixed	/
2	Closed	Fixed	Fixed	Vision
3	Open	Fixed	Sway-referenced	Vision
4	Open	Sway-referenced	Fixed	Somatosensory
5	Closed	Sway-referenced	Fixed	Somatosensory, vision
6	Open	Sway-referenced	Sway-referenced	Somatosensory, vision

*Sway-referenced means that the tilt of the platform and/or visual surround was followed directly to the anterior–posterior sway of the participant. “/” means no interference.*

##### Unilateral Stance

The unilateral stance (US) test required participants to stand on the side of the unstable ankle for 10 s. Their hands were on the anterior superior spine, and non-stance leg bent to ∼30° of knee flexion. Each trial was repeated three times, respectively, with eyes open and eyes closed.

##### Motor Control Test

Motor control test (MCT) quantified the effectiveness of automatic postural motor responses to unexpected forward/backward translations of the platform. The amplitudes of translations were scaled to the participant’s height at a constant speed, creating body sway in units of angular momentum [small (2.8°/s), medium (6.0°/s), and large (8.0°/s)]. The amplitude and directions of translations were randomly assigned. Each translation was repeated three times in succession with random intervals.

##### Adaption Test

Similar to MCT, adaption test (ADT) evaluated the ability to alter motor responses and minimize body sway during externally triggered perturbation. The platform would rotate suddenly at a velocity of 20°/s in the toes up or toes-down direction. Each rotation was repeated five times in succession with random intervals.

#### Data Analysis

Electromyography raw data were imported in the LabVIEW software (ProEMG, Myon AG, Schwarzenberg, Switzerland). They were filtered (dual-pass second order Butterworth, 20–500 Hz), full-wave rectified and smoothed with root mean square (RMS) smoothing window of 100 ms. For each muscle, the threshold for activation was defined as “all channels go above 5 × baseline noise standard deviations for at least 50 ms.” If not, the muscle was considered inactive. The RMS of all muscles was calculated for CDP trials. Maximum voluntary isometric contraction (MVIC) tests for each muscle were performed for five consecutive seconds according to the standard methods (Tabard-Fougère et al., 2018). Considering VM as an example, the participant short sat with a pad under the distal thigh to maintain the horizontal position of the femur. The hands rested on the table on either side of the body for stability. Their knee was flexed at approximately 30°, while giving verbal encouragement, resistance was applied in the direction of knee flexion. The participant was instructed to try their maximal force to extend the knee for 5 s (Tabard-Fougère et al., 2018). Then, RMS values were normalized with MVIC by calculating the percent activation (%MVIC) (Eq. 2). Postural reaction time latency measurements derived from the MCT on the NeuroCom EquiTest were quantified as the time period in milliseconds (ms) between the onset of the translation and the initiation of the participant’s active response.


(2) $$\mathrm{NEMG}=\frac{{\text{EMG}}_{\text{RMS}}}{{\text{MVIC}}_{\text{RMS}}}\times 100\%$$

#### Kinesthesia

The kinesthesia of the unstable ankle was determined using the threshold to the detection of passive motion direction with good reliability (Sun et al., 2015; Lai et al., 2018). Participants were instructed to sit on a chair, with both legs supported by the frames. By removing half of the weight of the lower limb, irrelevant sensory information would be reduced.

Prior to testing, participants must close their eyes and wear headphones. After performing two practice trials, a formal data collection was conducted. The platform randomly rotated toward one of the four directions (plantarflexion, dorsiflexion, inversion, and eversion of the ankle) at a rate of 0.4°/s with a random time interval between 2 and 10 s. Once the participants detected ankle movement, they would press a handheld stop button and confirm the direction of the motion. The rotation angles of the platform were determined as the threshold to detection of passive motion direction of the ankle. If participants identified the wrong direction, this trial shall be regarded as a failure, and the next test shall be carried out until three successful trials in each direction were recorded.

### Statistics Analysis

Data were expressed as mean ($\bar{x}$) and SD (*s*) and analyzed using SPSS statistical software (version 20.0, Chicago, IL, United States). The normal distribution of data was confirmed using the Shapiro–Wilk test. To determine differences among KT, AT, ST, and NT, one-way repeated measures ANOVA was conducted. Results were considered significant for *p* < 0.05. Bonferroni test was used for *post hoc* analysis. Effect size (ES) was reported using partial eta squared (η*_*p*_*^2^): small effect with 0.01 ≤ η*_*p*_*^2^ < 0.06, moderate effect with 0.06 ≤ η*_*p*_*^2^ < 0.138, and large effect with η*_*p*_*^2^ ≥ 0.138 (Cohen, 1988).

## Results

**FIGURE 2:**
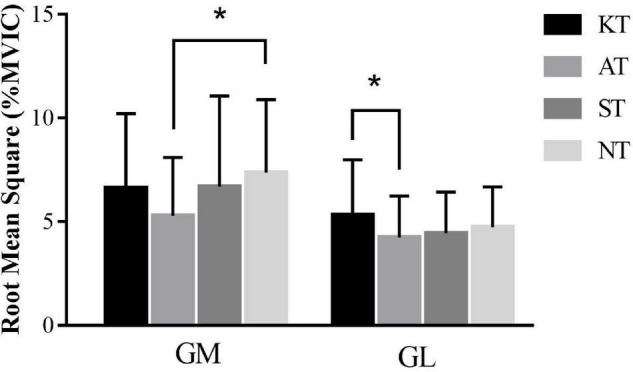
Comparison of root mean square values (%MVIC) in condition 4 during sensory organization test for gastrocnemius medialis and gastrocnemius lateralis. *means significant difference during different taping treatments.

During SOT, significant differences were observed for RMS values (%MVIC) as follows: for GM [*p* = 0.036, *F*(3,32) = 2.982, η*_*p*_*^2^ = 0.096] and for GL [*p* = 0.033, *F*(3,32) = 3.420, η*_*p*_*^2^ = 0.033] in condition 4. Specifically, GM RMS was 28.19% lower in AT compared with NT (*p* = 0.021, 95% CI = 0.002–0.039), while GL RMS was 20.25% lower in AT compared with KT (*p* = 0.038, 95% CI = 0.000–0.021) (Figure 2).

During the US, the results showed no significant difference in the four taping treatments for RMS values (%MVIC) of all examined muscles.

**FIGURE 3:**
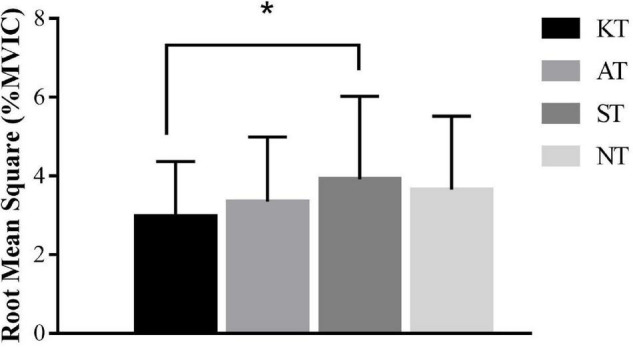
Comparison of root mean square values (%MVIC) in forward-small translation during motor control test for peroneal longus. * means significant difference during different taping treatments.

During forward-small translation of MCT, we found a significant difference [*p* = 0.022, *F*(3,32) = 3.412, η*_*p*_*^2^ = 0.116] for the RMS values (%MVIC) of PL. *Post hoc* analysis showed that PL RMS was 24.04% lower in KT compared with ST (*p* = 0.036, 95% CI = 0.000–0.018) (Figure 3). In addition, no significant difference for latencies was found among four taping treatments.

**FIGURE 4:**
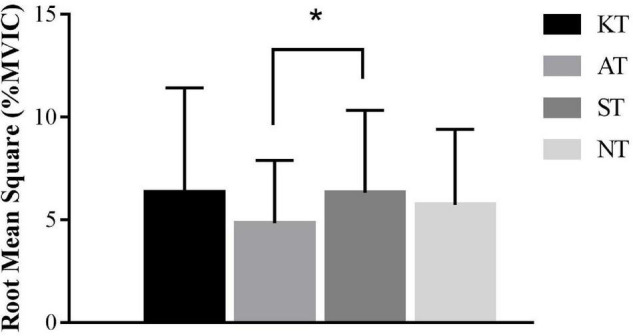
Comparison of root mean square values (%MVIC) in toes-down rotation during adaption test for peroneal longus. *means significant difference during different taping treatments.

During the toes-down rotation of ADT, significant difference was found among the four taping treatments [*p* = 0.022, *F*(3,32) = 3.356, η*_*p*_*^2^ = 0.098] for PL. Specifically, PL RMS was 23.41% lower in AT compared with ST (*p* = 0.015, 95% CI = 0.002–0.027) (Figure 4).

Regarding ankle kinesthesia, no significant difference was found for the threshold to the detection of passive motion direction among different taping treatments in ankle plantarflexion, dorsiflexion, inversion, and eversion.

## Discussion

The primary purpose of the study was to investigate the effects of KT, compared with non-elastic tape, ST, and no tape (NT), on muscle activation of the lower limb during CDP postural control tasks and ankle kinesthesia in individuals with CAI.

### Effect of Kinesiology Tape on Muscle Activation of the Lower Extremity During Computerized Dynamic Posturography Tasks

During SOT, contrary to the hypothesis, for the majority of conditions, no significant difference was found in the RMS values (%MVIC) in all examined muscles for different taping treatments during CDP tests. Just in condition 4, GM RMS was 28.19% lower in AT compared with NT (*p* = 0.021, 95% CI = 0.002–0.039), while GL RMS was 20.25% lower in AT compared with KT. Since the effect sizes were moderate, it was reasonable to conclude that the effect is statistically and clinically meaningful at a moderate level (Peterson and Foley, 2021). During condition 4, eyes were open, and the visual surround was fixed. Somatosensory information was interfered with by rotation of the platform along the ankle joint axis in proportion to spontaneous anterior–posterior sway. The triceps surae were compelled to contract in order to complete the posture regulation of ankle strategy. Under these circumstances, AT may provide greater ankle joint stability (Briem et al., 2011) to compensate for the blocking of proprioceptive input and subsequently decrease muscle activation of triceps surae. However, the effect was not observed in other conditions, indicating that KT and AT had a minor effect on lower limb muscle activation during self-initiated triggered perturbations. Therefore, practitioners must be more cautious for KT application in treating patients with CAI to face daily life and sports activities.

During the US, no significant difference was found among the four taping treatments for RMS values (%MVIC) of lower limbs with eyes open and eyes closed. The proposed mechanisms of KT are to increase the stimulation of cutaneous afferent and thus lead to muscle activation gain during single-leg stance. However, it seems that KT has no positive effect on lower limb muscle activation during the US. The reason for this phenomenon could be the taping technique that we selected did not cover the surface of the large muscles of the lower limbs and could not provide enough interaction between the muscles and KT (Fayson et al., 2015). In line with our results, Kodesh and Dar (2015) indicated that KT did not affect fibularis muscle activation during single-leg stance following ankle muscle fatigue compared with ST. However, other study demonstrated that KT decreased muscular activity amplitudes of peroneus longus muscle during single-leg stance (Sarvestan et al., 2020). Therefore, future research should pay more attention to the location and area covered by tape.

During MCT, no significant difference was found among different taping treatments for RMS values (%MVIC) of examined muscles during different translations. Except for PL, RMS was 24.04% lower in KT compared with ST during forward-small translation. The effect size was moderate, indicating KT had a moderate effect on decreasing the activation of PL (Peterson and Foley, 2021; Sharma, 2021). According to the hypothesis by Kase et al. (2003), taping would increase muscle activation through increased stimulation of cutaneous mechanoreceptors. This proposition is exactly the opposite of our observation. This phenomenon could be explained by the mechanical properties of tape, which could compensate for the ligament laxity and improve ankle joint stability (Sarvestan et al., 2020). On this basis, the nervous system may perceive increased support and adapt subsequently muscle recruitment to account for the decreased need for dynamic postural stability. However, this effect was not extended to other conditions during MCT, inspiring physiotherapists, in face of sudden center of gravity anterior–posterior imbalance during daily life, it seemed that KT had not enough effect to help CAI patients restore the center of gravity stability by activating lower limb muscles.

Postural response latencies to surface perturbation will determine the outcome of an induced loss of balance, and faster response latencies will induce faster balance response strategies. Our results align with a previous study (De-La-Morena et al., 2015) by demonstrating no significant difference among different taping methods.

Regarding ADT, we just found for PL, RMS was 23.41% significantly lower in AT compared with ST during sudden toes-down rotation. Even if there was a statistical difference, the effect size was small, suggesting that taping treatment had no practical clinical significance in unstable lower limb muscle activation during unexpected externally triggered toes-down rotation perturbations (Page, 2014; Peterson and Foley, 2021). Unlike our results, Briem et al. (2011) found that KT did not alter muscle activation during a sudden inversion perturbation, whereas AT could significantly increase muscle activation of fibularis longus. They explained that faced with unexpected inversion of the talocalcaneal joint, AT may provide enough support, which reduced the required contribution to the PL muscle (Fayson et al., 2015).

### Effect of Kinesiology Tape on Ankle Kinesthesia

As for kinesthesia, the results found no significant difference for the threshold to the detection of passive motion direction in dorsiflexion, plantarflexion, inversion, and eversion of the unstable ankle, indicating that KT has no positive effect in kinesthesia. This may be due to the elastic retraction force stimulation produced by KT being only limited to superficial skin, and reaching the proprioceptive receptors in the deep layer is difficult (Wang et al., 2019). Unlike our results, the previous study indicated that KT could provide sensory input to achieve greater ankle proprioceptive improvement (Simon et al., 2014; Long et al., 2017; Yu et al., 2021) for CAI.

### Clinical Implications

During CDP tasks, a variety of visual, somatosensory, and vestibular self-initiated perturbations and externally triggered perturbations were provided to simulate various situations in daily life. Unfortunately, it seems that KT had minimal effect on the muscle activation of the unstable lower limb to restore postural stability. Combined with small effect sizes, it could provide an inspiration to physiotherapists that the benefit of KT was too small to be clinically worthwhile over ST/NT while dealing with CAI patients (Peterson and Foley, 2021). Additionally, KT made no contribution to unstable ankle kinesthesia among CAI. Results of this study were consistent with previous meta-analysis (Parreira Pdo et al., 2014; Nunes et al., 2021), indicating current evidence was insufficient to support the use of KT in sports injury.

### Limitations

Limitations must be considered when interpreting data from this study. First, this study included male participants only. Thus, the results may not be applicable for women due to gender differences. Second, due to differences in neuromuscular control between dominant and non-dominant limbs (Del Bel et al., 2017), our study did not distinguish the consistency between unstable and dominant limbs; thus, this difference could be a confounding factor. Third, given that the posture stability adjustment strategy includes ankle strategy and hip strategy, we only measured the thigh and calf muscles, ignoring the muscle groups around the hip (gluteus maximus, gluteus medius, and iliopsoas). This study could not explore hip muscle activation. Finally, this study investigated the acute effect of KT application; hence, the results could not be extrapolated to extended effect.

## Conclusion

This study indicated that KT had minimal effect on muscle activation of the unstable lower limb during static stance, self-initiated, and externally triggered perturbation tasks from CDP and ankle kinesthesia among individuals with CAI. The results indicated the benefit of KT was too small to be clinically worthwhile while dealing with CAI for patients or physiotherapists. Nevertheless, the role of KT on an ankle injury and related mechanisms was still demanded to be explored to provide a more comprehensive evidence-based reference.

## Data Availability Statement

The raw data supporting the conclusions of this article will be made available by the authors, without undue reservation.

## Ethics Statement

The studies involving human participants were reviewed and approved by the Ethics Committee of Shanghai University of Sport. The patients/participants provided their written informed consent to participate in this study. Written informed consent was obtained from the individual(s) for the publication of any potentially identifiable images or data included in this article.

## Author Contributions

LY and KL contributed to recruiting the subjects, collecting the data, and writing the manuscript. CL undertook the statistical analysis. XF conceived of the study. LW interpreted the results. All authors contributed to the article and approved the submitted version.

## Conflict of Interest

The authors declare that the research was conducted in the absence of any commercial or financial relationships that could be construed as a potential conflict of interest.

## Publisher’s Note

All claims expressed in this article are solely those of the authors and do not necessarily represent those of their affiliated organizations, or those of the publisher, the editors and the reviewers. Any product that may be evaluated in this article, or claim that may be made by its manufacturer, is not guaranteed or endorsed by the publisher.
