# Supplementary material for: Effect of Kinesiology Tape on Muscle Activation of Lower Extremity and Ankle Kinesthesia in Individuals With Unilateral Chronic Ankle Instability
Source: Front Physiol. 2021 Dec 17;12:786584. doi: 10.3389/fphys.2021.786584 (PMC8718686; doi:10.3389/fphys.2021.786584)
Supplement: Supplementary file 1 [file Table_1.DOCX]

**SUPPLEMENTARY MATERIAL 1**

Counterbalanced randomization method is suitable for experiments conducted with repeated measures design. It aims to reduce the chances of the order of treatment influencing the results. During four taping treatments, taping of order followed:

|  | week1 | week2 | week3 | week4 |
| --- | --- | --- | --- | --- |
| Participant 1 | A | B | C | D |
| Participant 2 | A | B | D | C |
| Participant 3 | A | C | B | D |
| Participant 4 | A | C | D | B |
| Participant 5 | A | D | B | C |
| Participant 6 | A | D | C | B |
| Participant 7 | B | A | C | D |
| Participant 8 | B | A | D | C |
| Participant 9 | B | C | A | D |
| Participant 10 | B | C | D | A |
| Participant 11 | B | D | A | C |
| Participant 12 | B | D | C | A |
| …… | …… | …… | …… | …… |

A, KT; B,AT; C, ST; D,NT.
